# High expression of *BCL6* inhibits the differentiation and development of hematopoietic stem cells and affects the growth and development of chickens

**DOI:** 10.1186/s40104-020-00541-3

**Published:** 2021-02-05

**Authors:** Hongmei Li, Bowen Hu, Shang Hu, Wen Luo, Donglei Sun, Minmin Yang, Zhiying Liao, Haohui Wei, Changbin Zhao, Dajian Li, Meiqing Shi, Qingbin Luo, Dexiang Zhang, Qinghua Nie, Xiquan Zhang

**Affiliations:** 1 Department of Animal Genetics, Breeding and Reproduction, College of Animal Science, South China Agricultural University, Guangzhou, 510642 Guangdong Province China; 2 Guangdong Provincial Key Lab of AgroAnimal Genomics and Molecular Breeding and Key Lab of Chicken Genetics, Breeding and Reproduction, Ministry of Agriculture, Guangzhou, 510642 Guangdong China; 3 State Key Laboratory for Conservation and Utilization of Subtropical Agro-bioresources, South China Agricultural University, Guangzhou, 510642 Guangdong China; 4 Division of Immunology, Virginia-Maryland College of Veterinary Medicine, University of Maryland, College Park, MD USA

**Keywords:** *BCL6*, Hematopoietic stem cells, Mitochondrial function, Runting and stunting syndrome, Sex-linked dwarf chickens

## Abstract

**Background:**

B-cell CLL/lymphoma 6 (BCL6) is a transcriptional master regulator that represses more than 1200 potential target genes. Our previous study showed that a decline in blood production in runting and stunting syndrome (RSS) affected sex-linked dwarf (SLD) chickens compared to SLD chickens. However, the association between *BCL6* gene and hematopoietic function remains unknown in chickens.

**Methods:**

In this study, we used RSS affected SLD (RSS-SLD) chickens, SLD chickens and normal chickens as research object and overexpression of *BCL6* in hematopoietic stem cells (HSCs), to investigate the effect of the *BCL6* on differentiation and development of HSCs.

**Results:**

The results showed that comparison of RSS-SLD chickens with SLD chickens, the *BCL6* was highly expressed in RSS-SLD chickens bone marrow. The bone marrow of RSS-SLD chickens was exhausted and red bone marrow was largely replaced by yellow bone marrow, bone density was reduced, and the levels of immature erythrocytes in peripheral blood were increased. At the same time, the hematopoietic function of HSCs decreased in RSS-SLD chickens, which was manifested by a decrease in the hematopoietic growth factors (HGFs) EPO, SCF, TPO, and IL-3, as well as hemoglobin α_1_ and hemoglobin β expression. Moreover, mitochondrial function in the HSCs of RSS-SLD chickens was damaged, including an increase in ROS production, decrease in ATP concentration, and decrease in mitochondrial membrane potential (ΔΨm). The same results were also observed in SLD chickens compared with normal chickens; however, the symptoms were more serious in RSS-SLD chickens. Additionally, after overexpression of the *BCL6* in primary HSCs, the secretion of HGFs (EPO, SCF, TPO and IL-3) was inhibited and the expression of hemoglobin α_1_ and hemoglobin β was decreased. However, cell proliferation was accelerated, apoptosis was inhibited, and the HSCs entered a cancerous state. The function of mitochondria was also abnormal, ROS production was decreased, and ATP concentration and ΔΨm were increased, which was related to the inhibition of apoptosis of stem cells.

**Conclusions:**

Taken together, we conclude that the high expression of *BCL6* inhibits the differentiation and development of HSCs by affecting mitochondrial function, resulting in impaired growth and development of chickens. Moreover, the abnormal expression of *BCL6* might be a cause of the clinical manifestations of chicken comb, pale skin, stunted growth and development, and the tendency to appear RSS in SLD chickens.

## Background

B-cell CLL/lymphoma 6 (BCL6) is known as a carcinogenic driver. The protein of *BCL6* has two functional domains, including the hydrophobic BTB/POZ region at the N-terminal and the functional domain of zinc-finger structures that have homology with members of the C-terminal Kruppel subfamily of zinc-finger proteins [1]. BCL6 specifically binds to the promoter DNA of the target gene by the zinc-finger domain, thereby inhibiting the transcription of target genes. At present, research on *BCL6* mainly focuses on the structure formation of the B-cell germinal center, because BCL6 is also closely related to diffuse large B-cell lymphoma (germinal center type), and is thought to be a target for the treatment of cancer and autoimmune diseases.

Hematopoietic stem cells (HSCs) are primitive cells with the potential for self-renewal and multipolarity. Most HSCs are dormant, and only a small number can self-renew and differentiate into other types of daughter cells, which are important for maintaining the stability of the HSC bank and blood system *in vivo* [2]. Thus, under normal circumstances, most HSCs are in the non-proliferative G_0_ phase [3]. When HSCs and early hematopoietic progenitor cells are stimulated or positively regulated, or when the balance between the stimulus and the cytokine is disturbed, HSCs begin to proliferate [4].

In vertebrates, most blood production occurs in the bone marrow. HSCs exist in specific microenvironmental niches in the bone marrow. Bone marrow mesenchymal stem cells (MSCs) can support HSCs and provide various hematopoietic cytokines, which build a good spatial environment for the proliferation, differentiation, and maturation of HSCs. Hemopoietic growth factors (HGFs) mainly bind to related receptors, and transmit growth and differentiation signals, to regulate the survival and proliferation of hematopoietic stem/progenitor cells and to stimulate their differentiation, maturation, and release into various blood cells [5]. Previous studies have shown that bone marrow MSCs secrete various hematopoietic cytokines, including FIT3 ligands, TPO, SCF, G-CSF, GM-CSF, IL-3, IL-6, and LIF [6].

Moreover, most dormant HSCs are exposed to low oxygen levels, which may inhibit the proliferation of HSCs and maintain the dormant state of HSCs. In fact, even HSCs in the peripheral circulation show a hypoxia spectrum [7]. When HSCs are in a hypoxic state during the dormant period, low metabolic levels are maintained mainly by cytoplasmic glycolysis. Once the HSCs are activated into the proliferation and differentiation stage, they need the mitochondrial tricarboxylic acid cycle to produce large amounts of energy, because at this time the consumption of oxygen greatly increases [8]. Therefore, the energy metabolism of HSCs can be analyzed to predict their cell state. To date, the association between the *BCL6* gene and hematopoietic function still remains unknown in chickens.

Sex-linked dwarf (SLD) chickens are caused by mutations or deletions in the *GHR* gene, which are small in size (only 60–70% of normal chicken weight). They are particularly prone to appear runting and stunting syndrome (RSS) [9], which is characterized by low body weight, generally occurs early in life, and leads to considerable economic losses in the commercial broiler industry [10]. In a previous study, we observed a decline in blood production in RSS affected SLD (RSS-SLD) chickens compared to SLD chickens [9]. In this study, we used RSS-SLD chickens, SLD chickens and normal chickens as research object and overexpression of the *BCL6* in HSCs, to study the effect of the *BCL6* on differentiation and development of chicken HSCs.

## Methods

### Ethics statement

All animal experiments were performed according to the protocols approved by the South China Agriculture University Institutional Animal Care and Use Committee (approval number SCAU#0017). All animal procedures followed the regulations and guidelines established by this committee and minimized the suffering of animals.

### Chickens

The 140-day-old Cantonese-yellow chickens used in the living experiment were provided by the breeding farm of South China Agricultural University. The 14-day-old chickens used for hematopoietic stem cell isolation were provided by Zhejiang GD poultry Co., Ltd.

In all, 6 RSS affected SLD (RSS-SLD) chickens, 6 SLD chickens and 6 normal chickens at 140-day-old of age in strain Cantonese-yellow chickens were utilized to explore the molecular mechanism of the *BCL6* *in vivo* by regulating the differentiation and development of HSCs to affect the growth characteristics of chicken as previously described [9]. The 14-day-old normal chickens were only utilized for HSCs and MSCs isolation to study the function of the *BCL6* *in vitro*.

### Paraffin sections and HE staining

The samples of 140-day-old RSS-SLD chickens, SLD chickens and normal chickens were fixed with 10% neutral formalin for 5 days and then immersed in hydrochloric acid/formic acid working solution to complete the decalcification. After decalcification, the samples were dehydrated in alcohol and transformed into a transparent state using xylene. After the transparency step was completed, the samples were soaked in wax and embedded in paraffin. A paraffin sectioning machine was used to cut 7–10 μm sections, which were stained with hematoxylin and eosin.

### Red blood cell count

Blood samples of 140-day-old RSS-SLD chickens, SLD chickens and normal chickens were diluted, and the number of red blood cells was calculated using a blood cell count board.

### Hematocrit determination

Blood samples of 140-day-old RSS-SLD chickens, SLD chickens and normal chickens were collected into tubes containing EDTA, which was drawn into a capillary tube that was sealed with glue at the bottom. Each sample was centrifuged at 3000 r/min for 30 min, the blood is divided into three layers. The ratio of the bottom red blood cell layer to the whole cell column was calculated and taken as the level of hematocrit.

### Blood smear and Wright-Giemsa staining

Each blood sample of 140-day-old RSS-SLD chickens, SLD chickens and normal chickens was uniformly coated on a slide and allowed to dry naturally. Then two to three drops of Wright-Giemsa dye were added to completely cover the specimen and the sample was subjected to staining for 1–2 min. Next, the same amount of phosphate buffer as the dye (pH 6.4) was added, the sample was shaken slowly, and the staining was allowed to proceed for another 3–5 min. The whole process must keep the sample moist to prevent pigment deposition. Finally, the sample was washed, dried, and inspected under an optical microscope (Leica, Germany).

### Quantitative real-time PCR

RNA was extracted from tissues or cells using RNAiso reagent (Takara, Japan) according to the manufacturer’s protocol. The concentration of RNA samples and OD value of 260/280 were detected using a Nanodrop 2000c spectrophotometer (Thermo, USA). Samples were stored at − 80 °C for later use. cDNA was synthesized using a PrimeScript RT Reagent Kit (Takara, Japan) for quantitative real-time PCR (qRT-PCR). The MonAmp™ ChemoHS qPCR Mix (Monad Co., LTD, Guangzhou, China) was used for qRT-PCR using a Bio-Rad CFX96 Real-Time Detection instrument (Bio-Rad, USA) according to the manufacturer’s protocol. The reaction procedure included initial denaturation at 95 °C for 3 min, followed by denaturation at 95 °C for 10 s, annealing at 60 °C for 20 s, extension at 72 °C for 10 s, for a total of 40 cycles. At the end of the cycle, the dissolution curve was analyzed and the detection temperature was 70–95 °C. Relative gene expression was measured using qRT-PCR twice for each reaction, and *GADPH* was used as a control. The primers used for qRT-PCR are listed in Table 1.

**Table 1:** Primer sequences of qRT-PCR

Genes	Primer sequences (5'→3')	Temperature, °C	Size, bp
*BCL6*	GCAGTTCAGAGCCCACAAAA	58	205
	GTTCAGACGGGAGGTGTACA		
Hemoglobin α_1_	GTCAACTTCAAACTCCTGGGC	57	140
	TAACGGTACTTGGCGGTCAG		
Hemoglobin β	AGAACTTCAGGCTCCTGGGTG	57	167
	GTGCTCCGTGATCTTTGGTG		
*GAPDH*	TCCTCCACCTTTGATGCG	50–65	225
	GTGCCTGGCTCACTCCTT		

### Extraction of peripheral blood HSCs, bone marrow HSCs, and MSCs

Peripheral blood HSCs, bone marrow HSCs, and MSCs were extracted using the appropriate separation kits (TBDscience, China) following the manufacturer’s protocol.

### Cell culture

HSCs and MSCs were cultured in Iscove’s Modified Dulbecco’s Medium (Gibco, USA) with 15% fetal bovine serum (ExCell Bio, China) and 0.2% penicillin/streptomycin (Invitrogen, USA). All cells were cultured at 37 °C in a 5% CO_2_ humidified atmosphere.

### Transfection

Cells were plated onto a culture plate and incubated overnight prior to the transfection experiment. Transfection was performed using Lipofectamine 3000 reagent (Invitrogen, USA) following the manufacturer’s protocol, and nucleic acids were diluted in OPTI-MEM Medium (Gibco, USA). All cells were analyzed 48 h after transfection.

### Plasmid construction

The plasmid pcDNA3.1-*BCL6* was generated by amplifying *BCL6* coding sequences from RNA by RT-PCR, and then subsequently cloning them into the pcDNA3.1 vector (Promega, USA) using a pMD18-T cloning vector (Takara, China) with the *EcoRI* and *HindIII* restriction sites. Plasmid constructs were confirmed by Sanger sequencing. The primers utilized for vector construction were showed in Table 2 and synthesized by Sangon Biotech (Shanghai, China).

**Table 2:** Primer amplification of target gene *BCL6* CDS region

Primers	Primer sequences (5'→3')	Size, bp
*BCL6*-CDS-F	CCGGAATTCATGGCCTCACCGGCAGACAGCTGC	2127
*BCL6*-CDS-R	CCCAAGCTTTCAGCAAGCCTTGGGGAGCTC	

### Detection of reactive oxygen species

The production of reactive oxygen species (ROS) in the mitochondria of cells was measured using an ROS assay kit (Beyotime, China) according to the manufacturer’s protocol. Dichlorofluorescein (DCF) fluorescence was determined using a Fluorescence/Multi-Detection Microplate Reader (BioTek, USA) as previously described [11]. Data were normalized to the control group and are expressed as a percentage of control levels.

### Detection of ATP content

ATP levels were measured using an ATP assay kit (Beyotime, China) according to the manufacturer’s protocol. A Fluorescence/Multi-Detection Microplate Reader (BioTek, USA) was used to determine ATP levels. Data were normalized to the control group and are expressed as a percentage of control levels.

### Detection of mitochondrial membrane potential

Mitochondrial membrane potential (ΔΨm) was measured using a JC-1 kit (Beyotime, China) according to the manufacturer’s protocol. The mitochondria were fixed with JC-1. The fluorescence was determined using a Fluorescence/Multi-Detection Microplate Reader (BioTek, USA) after the cells were incubated with JC-1 for 20 min at 37 °C; 10 μmol/L rotenone was used as a standard inhibitor of ΔΨm. The data (the ratio of aggregated and monomeric JC-1) were normalized to the control group and are expressed as a percentage of control levels.

### Cytokine levels determined by ELISA

ELISA was used to detect the content of various HGFs *in vivo* and *in vitro* according to the instructions of commercial ELISA kits (mlbio, China). In brief, the plasma of 140-day-old chickens with EDTA and cell culture supernatant were centrifuged at 4000×*g* for 5 min at 4 °C. Then, the supernatant of 10 μL samples was collected and added to the bottom of the plate well with 40 μL sample diluents along with 100 μL enzyme-labeled reagents. After the plate was sealed with the sealing film, the plate was incubated at 37 °C for 60 min. Finally, we performed washing, color rendering and termination of the plate following standard procedures. Absorbance at 450 nm was determined using a Fluorescence/Multi-Detection Microplate Reader (BioTek, USA).

### Statistical analyses

All experiments were performed at least three times. The data are presented as means ± standard error of the mean (SEM). Statistical analyses were performed using Student’s *t*-test, and we considered *P* < 0.05 to be statistically significant, *P < 0.05, **P < 0.01, ***P < 0.001.

## Results

### Erythrocyte specific volume and erythrocyte count *in vivo*

The results showed that red blood cell count of RSS-SLD chickens and SLD chickens was higher than that of normal chickens, but the difference between RSS-SLD chickens and SLD chickens was not significant (Table 3). Hematocrit can indirectly reflect the number and volume of erythrocytes, and we found that RSS-SLD chickens and SLD chickens had more hematocrit than normal chickens, the results were consistent with the red blood cell count (Table 3).

**Table 3:** Determination of erythrocyte specific volume (PCV) and erythrocyte number

Groups	Number	PCV, %	Blood red cell count, × 10^9^/mL
RSS-SLD	6	46.708 ± 0.005^a^	1.130 ± 0.018^a^
SLD	6	46.488 ± 0.025^a^	1.028 ± 0.099^a^
Normal	6	42.997 ± 0.007^b^	0.995 ± 0.067^b^

^a, b^Within a column, values not sharing a common superscript are significantly different

### Peripheral blood smears

**Fig. 1:**
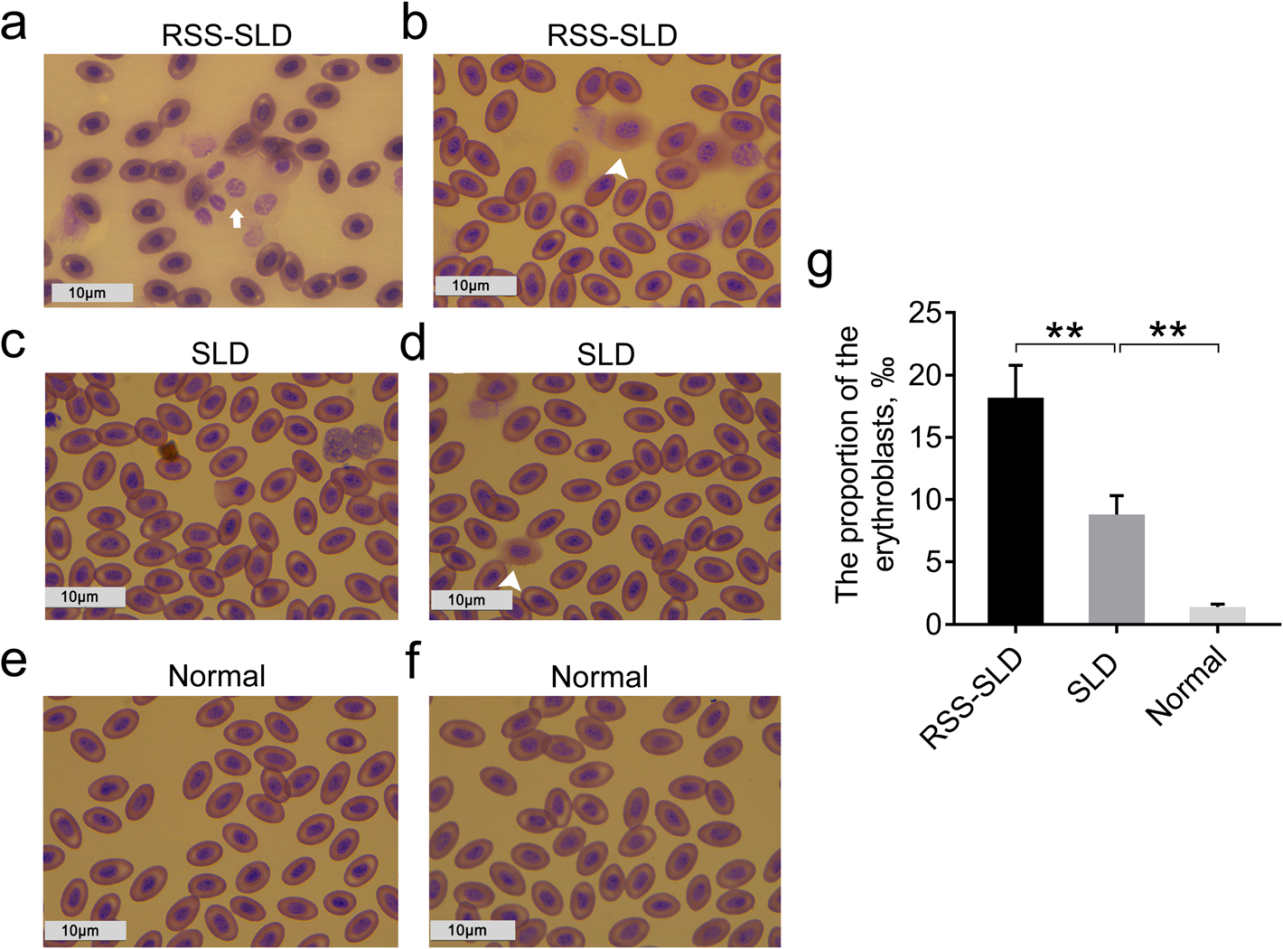
Results of peripheral blood smears in 140-day-old RSS-SLD chickens, SLD chickens and normal chickens. **a** and **b** are the peripheral blood smears of SLD-RSS chickens; bar 10 μm; **c** and **d** are the peripheral blood smears of RSS chickens; bar 10 μm; **e** and **f** are the peripheral blood smears of normal chickens; bar 10 μm; **g** The statistical of the proportion of the erythroblasts in the peripheral blood smears of RSS-SLD, SLD chickens and normal chickens. Data are expressed as means ± SEM, **P < 0.01

Figure 1a-f showed that a large number of erythroblasts at various stages in the peripheral blood of RSS-SLD chickens, and some cells were deformed with seriously damaged cell membranes. Erythroblasts were also present in SLD chickens, but this was not obvious comparison with RSS-SLD chickens. Furthermore, comparison of the RSS-SLD chickens with SLD chickens and SLD chickens with normal chickens revealed that the proportion of the erythroblasts (‰) in RSS-SLD chickens and SLD chickens was significantly increased, indicating that the differentiation and maturation of red blood cells was impaired in RSS-SLD chickens and SLD chickens (Fig. 1g).

### Bone marrow tissue section results

**Fig. 2:**
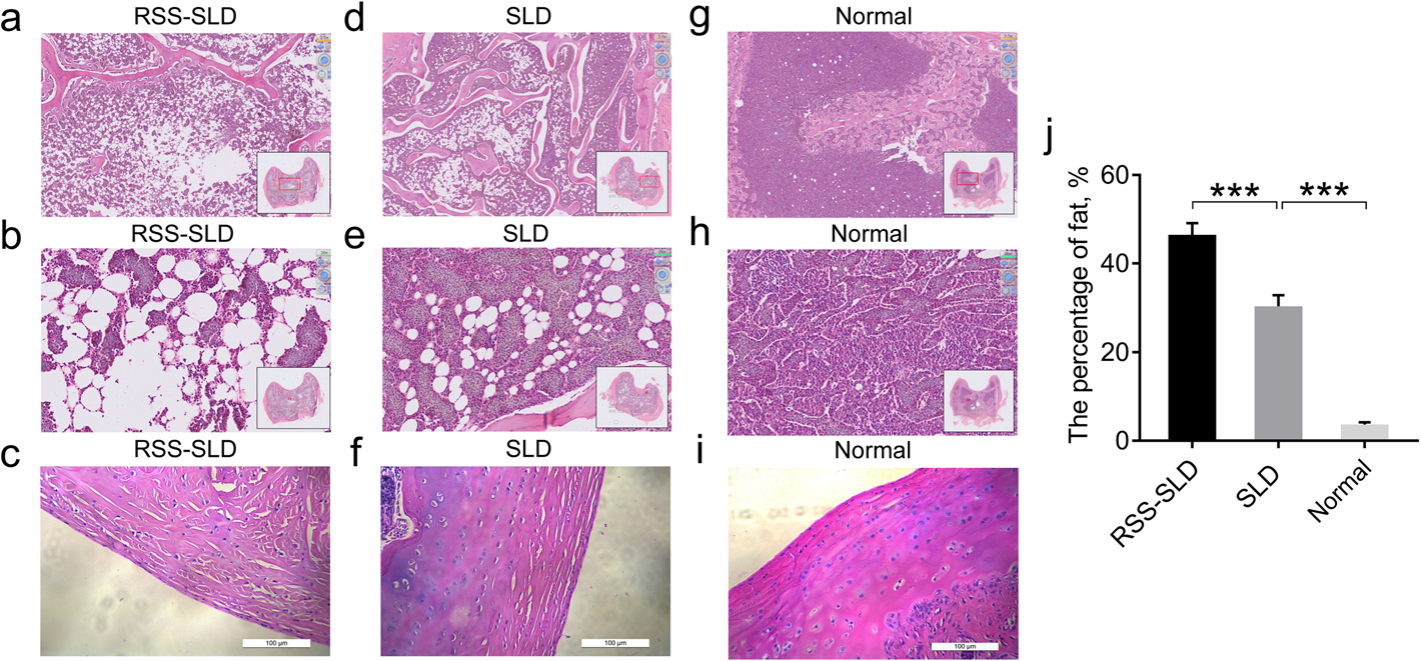
The bone marrow slices of 140-day-old RSS-SLD chickens, SLD chickens and normal chickens. **a** and **b** are the bone marrow sections of RSS-SLD chickens; **d** and **e** are the bone marrow sections of SLD chickens; **g** and **h** are the bone marrow sections of SLD chickens; **c**, **f** and **i** are the cartilage sections of RSS-SLD, SLD chickens and normal chickens respectively; bar 100 μm. **j** The percentage of fat in volume (%) of RSS-SLD, SLD chickens and normal chickens. Data are expressed as means ± SEM, ***P < 0.001

Paraffin sections of bone marrow showed that the ratio of yellow fat of RSS-SLD chickens was significantly higher compared with SLD chickens, and that of SLD chickens was significantly higher compared with normal chickens, indicating the bone marrow of SLD chickens was failure and this symptom was more serious in RSS-SLD chickens (Fig. 2a-i). Besides, Fig. 2c, f and i showed that large and small cracks appeared in the cartilage of both chicken types, but the symptoms were more serious in RSS-SLD chickens. This indicates that different degrees of chondral osteoporosis were observed in RSS-SLD chickens and SLD chickens, and that bone density was severely reduced. This might be the cause of the skeleton dysplasia, short stature, and easy to appear slope foot in SLD chickens.

### BCL6 content and hemoglobin expression in peripheral blood HSCs

**Fig. 3:**
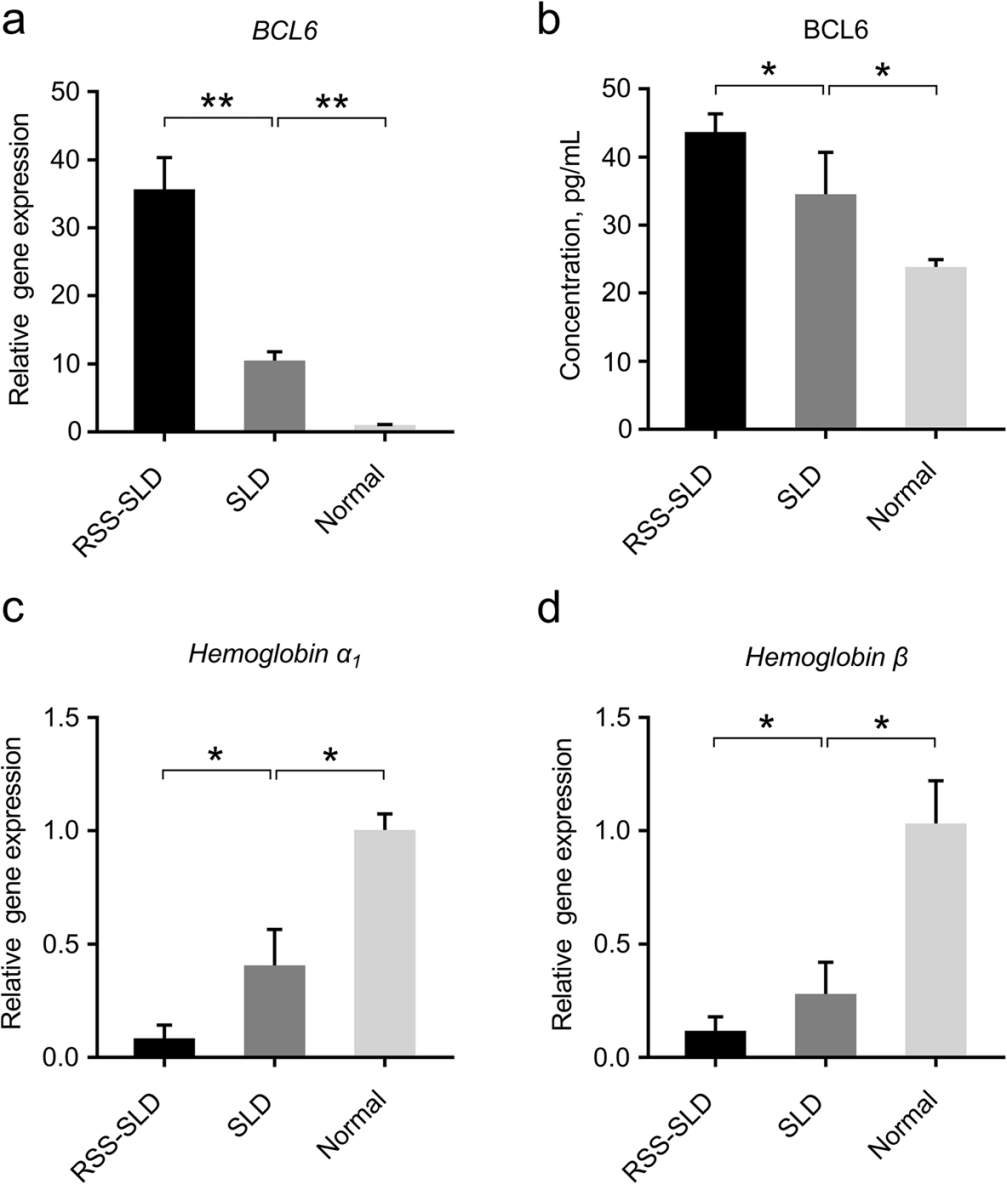
Relative expression levels of various genes in peripheral blood HSCs of 140-day-old RSS-SLD chickens, SLD chickens and normal chickens. **a** The HSCs were extracted from peripheral blood and the expression of *BCL6* was determined by qRT-PCR. **b** *BCL6* expression in peripheral blood plasma was determined by ELISA. **c** Expression of hemoglobin α_1_ in peripheral blood HSCs. **d** Expression of hemoglobin β in peripheral blood HSCs. Data are expressed as means ± SEM, *P < 0.05, **P < 0.01

Preliminary work using microarray expression profiling showed that *BCL6* expression was upregulated in all dwarf strains. In present study, we speculated that *BCL6* was related to the insufficient hematopoietic capacity of RSS-SLD chickens and SLD chickens. Therefore, we first explored the functionality of *BCL6*, and then measured the expression levels of hemoglobin α_1_ and hemoglobin β. The results showed that the *BCL6* expression of RSS-SLD chickens was significantly increased compared with SLD chickens, and that of SLD chickens was significantly increased compared with normal chickens (Fig. 3a). At the same time, we also detected the content of BCL6 via ELISA and confirmed that it was significantly higher in RSS-SLD chickens and SLD chickens (Fig. 3b). Moreover, the hemoglobin α_1_ and hemoglobin β expression of RSS-SLD chickens were significantly reduced compared to SLD chickens, and that of SLD chickens were significantly reduced compared to normal chickens (Fig. 3c, d).

### Determination of various HGFs in plasma by ELISA

**Fig. 4:**
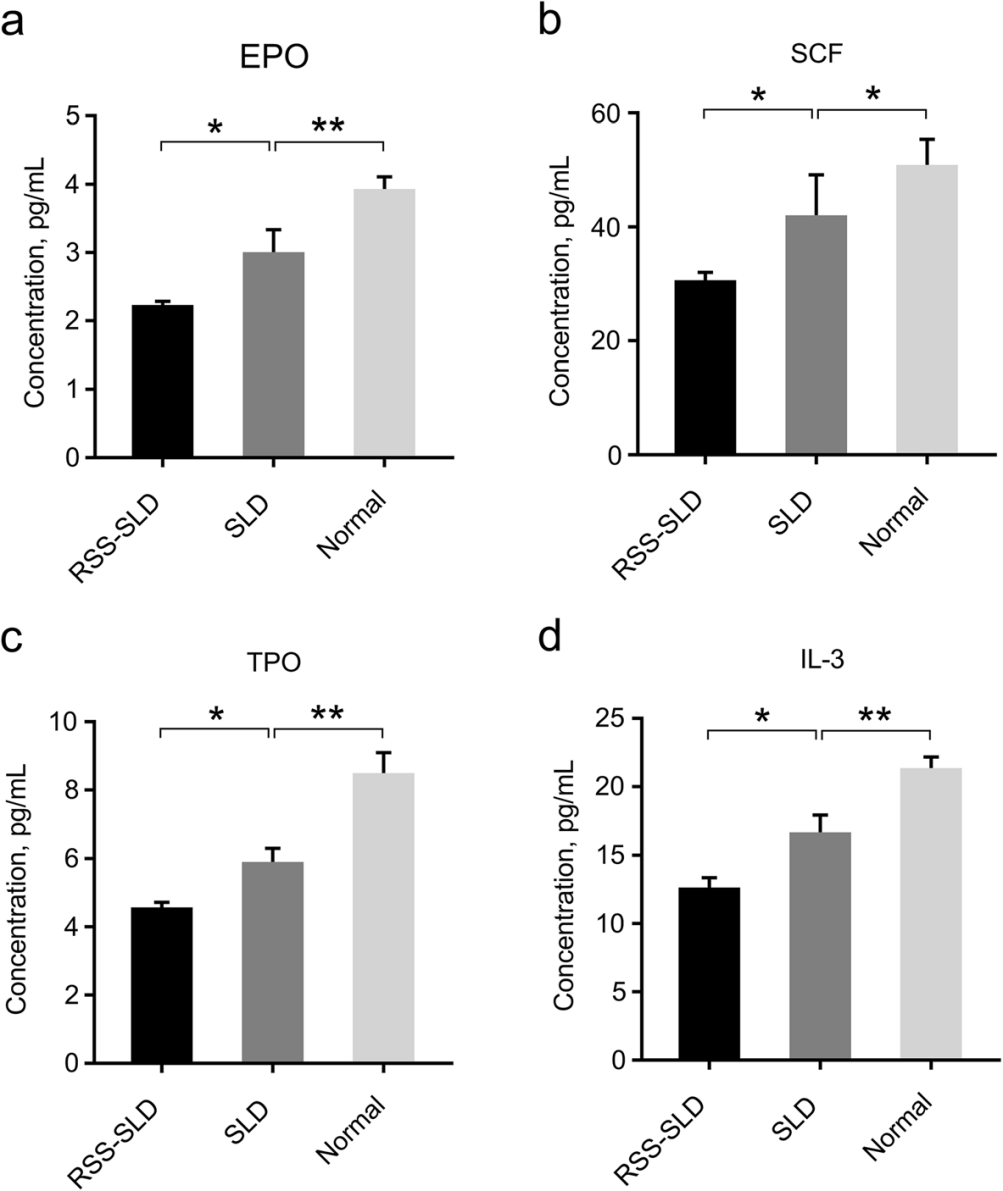
Determination of various HGFs of 140-day-old RSS-SLD chickens, SLD chickens and normal chickens in plasma by ELISA. **a** The EPO content in plasma was detected by ELISA. **b** The SCF content in plasma was detected by ELISA. **c** The TPO content in plasma was detected by ELISA. **d** The IL-3 content in plasma was detected by ELISA. Data are expressed as means ± SEM, *P < 0.05, **P < 0.01

HGFs play an important role in hematopoietic function. To reflect the hematopoietic function of RSS-SLD chickens, SLD chickens and normal chickens, we used ELISA to detect the levels of various HGFs in plasma. The concentrations of EPO, SCF, TPO, and IL-3 of RSS-SLD chickens were all significantly reduced compared to SLD chickens, and that of SLD chickens were all significantly reduced compared to normal chickens (Fig. 4a-d). These HGFs promote the differentiation and development of HSCs, and thus our results indicated that the differentiation and development of HSCs were inhibited in RSS-SLD chickens and SLD chickens.

### Detection of mitochondrial activity of HSCs in RSS-SLD chickens, SLD chickens and normal chickens

**Fig. 5:**
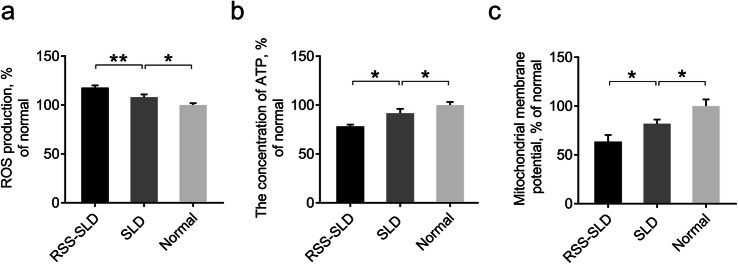
Detection of mitochondrial function of peripheral blood HSCs in 140-day-old RSS-SLD chickens, SLD chickens and normal chickens. **a** Detection of ROS levels of peripheral blood HSCs in RSS-SLD chickens, SLD chickens and normal chickens. **b** Detection of ATP levels of peripheral blood HSCs RSS-SLD chickens, SLD chickens and normal chickens. **c** Detection of the ΔΨm of peripheral blood HSCs RSS-SLD chickens, SLD chickens and normal chickens. Data are expressed as means ± SEM, *P < 0.05, **P < 0.01

Mitochondria provide energy for HSCs and maintain the hematopoietic microenvironment. Therefore, HSCs were extracted from the peripheral blood of the chickens to detect mitochondrial function. ROS production was significantly increased, and the ATP concentration along with ΔΨm was significantly reduced in RSS-SLD chickens’ HSCs compared with SLD chickens (Fig. 5a-c). The same results were also observed in SLD chickens’ HSCs compared with normal chickens, indicating that impaired mitochondrial function of HSCs in RSS-SLD chickens and SLD chickens might affect the differentiation and development of HSCs in these chickens (Fig. 5a-c).

### Effects of overexpression of the *BCL6* on two subunits of hemoglobin in HSCs

**Fig. 6:**
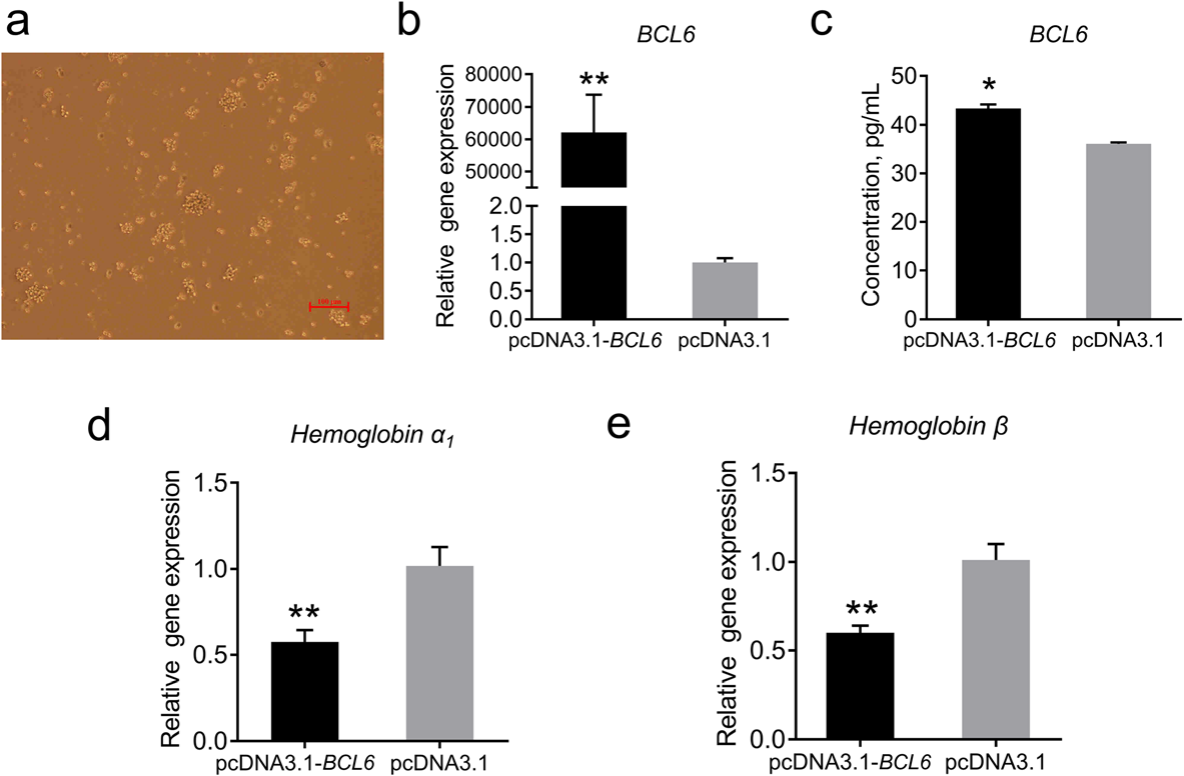
Morphology of HSCs and the overexpression efficiency of the *BCL6*. **a** Morphology of the HSCs; bar 100 μm. **b** Efficiency of the pcDNA3.1-*BCL6* vector overexpression. **c** *BCL6* expression in HSCs was determined by ELISA. **d** Expression of hemoglobin α_1_. **e** Expression of hemoglobin β. Data are expressed as means ± SEM, *P < 0.05, **P < 0.01

Next, we studied the effects of the *BCL6* on chicken HSCs *in vitro* (Fig. 6a). The expression of the *BCL6* was significantly increased, which was consistent with the ELISA results, indicating successful overexpression of the *BCL6* in HSCs (Fig. 6b, c). The expression of both subunits of hemoglobin (hemoglobin α_1_, hemoglobin β) were significantly decreased after *BCL6* overexpression, which was consistent with the *in vivo* results (Fig. 6d, e).

### Effects of overexpression of the *BCL6* on HGFs secretion by bone marrow MSCs

**Fig. 7:**
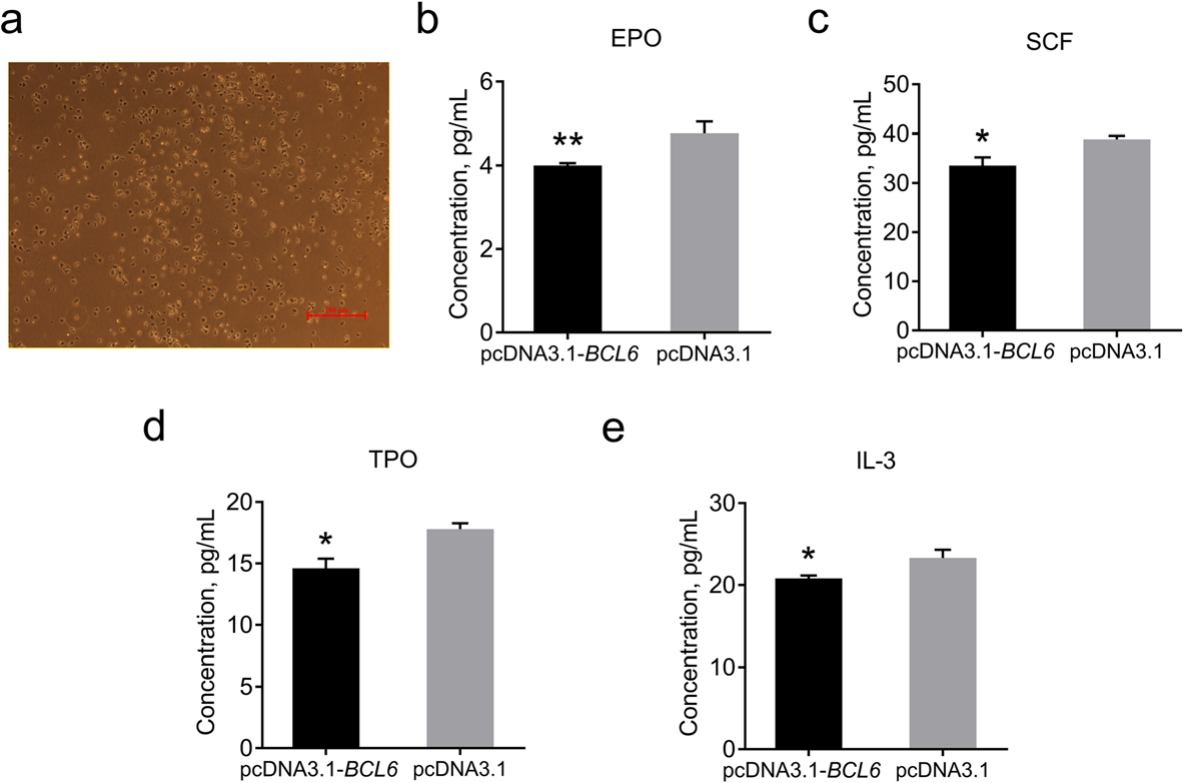
Morphology of bone marrow MSCs and contents of various HGFs. **a** Morphology of bone marrow MSCs; bar 100 μm. **b** Content of EPO levels after overexpression of *BCL6*. **c** Content of SCF levels after overexpression of the *BCL6*. **d** Content of TPO levels after overexpression of the *BCL6*. **e** Content of IL-3 levels after overexpression of the *BCL6*. Data are expressed as means ± SEM, *P < 0.05, **P < 0.01

Bone marrow MSCs not only provide support for HSCs, but also secrete HGFs to promote the differentiation of HSCs. Therefore, we overexpressed the *BCL6* in primary bone marrow MSCs. The bone marrow MSCs phenotype was identified by qRT-PCR verification, and *CD29*, *CD44*, *CD90*, and *CD71* were positive, while *CD31*, *CD34*, and *CD45* were negative. We also detected the secretion of various HGFs by ELISA (Fig. 7a). The concentrations of EPO, SCF, IL-3, and TPO were all significantly reduced after *BCL6* overexpression, indicating that the ability of bone marrow MSCs to secrete cytokines was inhibited (Fig. 7b-e).

### Effects of overexpression of the *BCL6* on HSCs proliferation and apoptosis

**Fig. 8:**
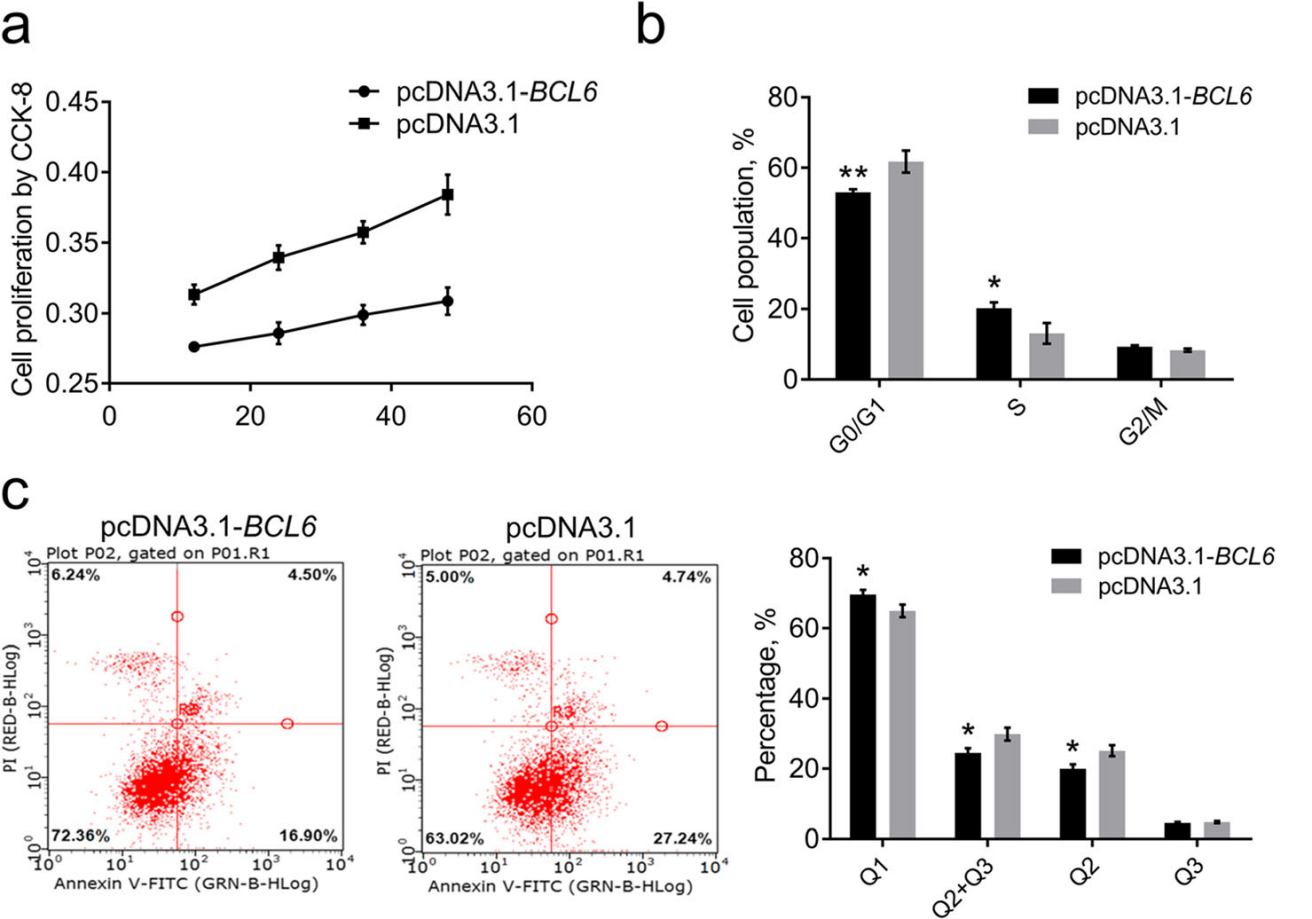
Effects of overexpression of the *BCL6* on HSCs proliferation and apoptosis. **a** Cell proliferation was detected by CCK8 after overexpression of the *BCL6* in HSCs. **b** The cell cycle was detected by flow cytometry after overexpression of the *BCL6* in HSCs. **c** Cell apoptosis was detected by flow cytometry after overexpression of the *BCL6* in HSCs. Data are expressed as means ± SEM, *P < 0.05, **P < 0.01

Next, we studied the effects of *BCL6* on HSCs proliferation and apoptosis. The overexpression of the *BCL6* promoted the proliferation of HSCs, as detected by CCK8 (Fig. 8a). Flow cytometry also showed that the percentage of HSCs in the G_0_/G_1_ phase significantly decreased after *BCL6* overexpression (Fig. 8b). However, the percentage of HSCs in the S phase (DNA synthesis) significantly increased (Fig. 8b). Furthermore, the percentage of HSCs in the G_2_/M phase did not significantly alter after *BCL6* overexpression (Fig. 8b). The G_2_ phase is the late stage of DNA synthesis; cell division begins in the M phase. This indicates that the overexpression of the *BCL6* promoted the proliferation of HSCs. Furthermore, we explored the effects of the *BCL6* on HSCs apoptosis and found that the proportion of HSCs in the Q_1_ phase (representing the percentage of living cells) was significantly increased after *BCL6* overexpression. Meanwhile, the proportion of HSCs in the Q_2_ phase (representing early apoptotic cells) was significantly reduced, and that in the Q_3_ phase (representing late apoptotic cells) did not significantly change after *BCL6* overexpression (Fig. 8c). These results demonstrated that overexpression of the *BCL6* inhibited the apoptosis of HSCs.

### Effects of overexpression of the *BCL6* on mitochondrial function of HSCs

**Fig. 9:**
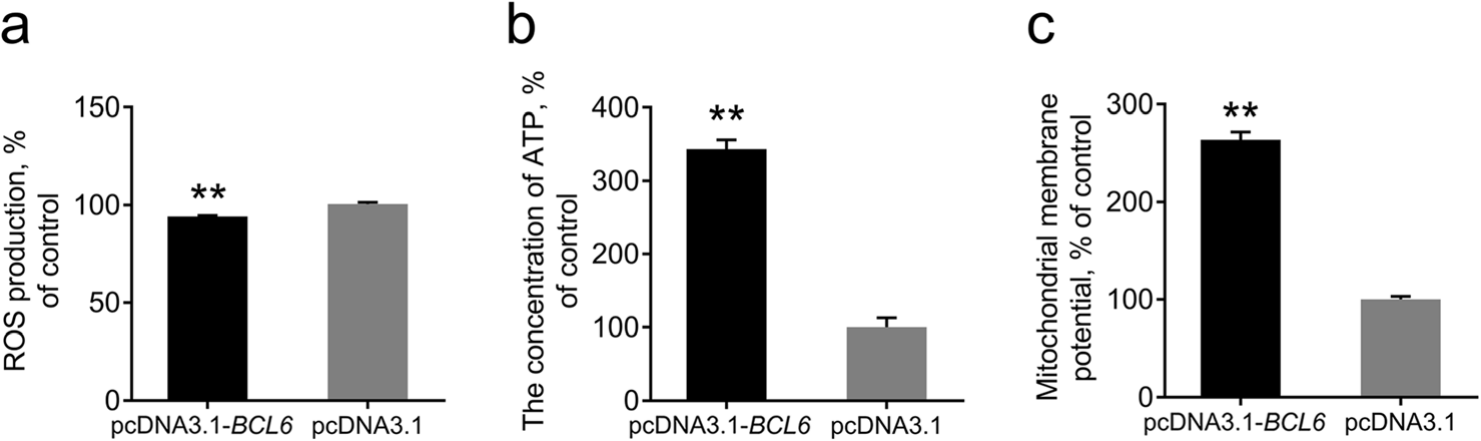
Effects of overexpression of *BCL6* on mitochondrial function in HSCs. **a** Detection of ROS levels in HSCs. **b** Detection of ATP levels in HSCs. **c** Detection of ΔΨm in HSCs. Data are expressed as means ± SEM, **P < 0.01

Finally, we detected various mitochondrial function indexes after overexpression of the pcDNA3.1-*BCL6* vector in HSCs. ROS production was significantly reduced, and the concentration of ATP along with ΔΨm was significantly increased after *BCL6* overexpression, indicating that the overexpression of the *BCL6* promoted mitochondrial function in HSCs (Fig. 9a-c).

## Discussion

RSS in chickens was first described in 1977, the cause of RSS chicken has not been clarified. At present, the research on the causes of RSS have mainly focused on pathogen infection, feeding management, and nutrition levels [10]. In a previous study on RSS-SLD chickens, we observed no inflammatory response or bacterial infection, no reticulum endothelial hyperplasia, subtype J avian leukemia, or Marek’s disease [9]. In this study, we studied the relationship between the hematopoietic disorder caused by the abnormal expression of *BCL6* and RSS in SLD chickens.

The red bone marrow of RSS-SLD chickens changed to yellow bone marrow prematurely, which made the bone marrow enter a pathological state. In the early stages of life, the bone is filled with red bone marrow, which is actively involved in hematopoietic function. Red bone marrow is the body’s hematopoietic organ, which can produce red blood cells, platelets, granulocytes, and other blood cells. In addition to the powerful hematopoietic function, red bone marrow has defense, immunity, wound repair, and other functions. Yellow bone marrow is mainly composed of fat, and hematopoiesis ability is weak. The transition from red to yellow bone marrow also signifies aging [12]. Therefore, the change from red to yellow bone marrow in RSS-SLD chickens indicates that the MSCs of these chickens differentiate more readily into fat, and that these chickens may show premature aging.

Accelerated aging of the body leads to significantly decreased function of the immune system (including B lymphocytes and T lymphocytes), gradual decreases in the number and quality of bone marrow cells and HSCs [12], and anemia [13]. It is also accompanied by a decrease in bone formation, lower bone density, and an increase in yellow fat in bone marrow. This is consistent with the symptoms of RSS-SLD chickens such as a pale comb, anemia due to insufficient hematopoiesis, stunting, low immunity, and lameness. Bone marrow exhaustion leads to the continuous loss of HSCs from the bone marrow niche, resulting in an increase in the number of red blood cells *in vitro*, which is also a reason for the increase in the number of red blood cells in the peripheral blood of RSS-SLD chickens. According to the results of peripheral blood smears, RSS-SLD chickens had juvenile red blood cells at various stages. Immature red blood cells, including early, middle, and late juvenile cells, are round to oval in shape, with basophilic cytoplasm and light pigmentation, and the nuclei are round to oval in shape with loose chromatin. As the cells mature, the density of chromatin gradually increases, and the cells shrink and become more oval. The abnormalities of red blood cells are mainly reflected as changes in size, number, color, shape, and nature, and such changes can reflect the anemia type, anemia degree, and bone marrow condition of the body [14]. We found that the peripheral blood of RSS-SLD chickens had a large number of young red blood cells at various stages. Moreover, some of the cells were malformed with seriously damaged membranes and low levels of hemoglobin. These results indicate that the hematopoietic function of the RSS-SLD chickens was seriously hindered, but do not reveal the specific mechanism involved.

To further investigate this issue, we examined the function of mitochondria, which affect the differentiation and development of HSCs. ROS levels were significantly higher in the HSCs of RSS-SLD chickens and SLD chickens. ROS are key chemicals in cells, which at controlled concentrations act as second messengers to mediate cellular responses to various endogenous and exogenous signals [15]. However, at high concentrations, they cause redox imbalance and oxidative stress [16]. An increase in ROS can also induce DNA damage. It also affects the copy number of mitochondrial DNA replications and leads to the decline of mitochondrial function [17]. Moreover, the damage of mitochondria will further promote the production of ROS. It is a vicious cycle. Increasing evidence suggests that oxidative stress, particularly ROS levels, affects the development, migration, and self-renewal of stem cells and the state of their cell cycle [18]. ATP levels were significantly lower in RSS-SLD chickens and SLD chickens, possibly due to the damaged mitochondrial structure caused by high ROS levels. A drop in ATP lowers the energy supply to the body, affecting muscle development and HSCs differentiation. This may be why RSS-SLD chickens are mostly listless. The main function of ΔΨm is to drive the synthesis of ATP through oxidative phosphorylation (OXPHOS). Therefore, the decrease in ΔΨm in RSS-SLD chickens will also affect the synthesis of ATP [19].

Moreover, various hematopoietic cytokines play an important role in the differentiation of HSCs into various daughter cells. They can regulate the survival and proliferation of HSCs and stimulate the differentiation, maturation, and release of HSCs into blood cells of different lines. The decrease in hematopoietic cytokines in RSS-SLD chickens resulted in the differentiation and maturation of stem cells. This is a reason for the decrease in hemoglobin α_1_ and hemoglobin β expression in red blood cells.

To further confirm our previous conjecture on the mechanism of low immunity and slow growth in RSS-SLD chickens and SLD chickens, we extracted bone marrow HSCs from chickens for cellular verification. The *BCL6* gene is an oncogene involved in B-cell lymphoma, which can drive malignant phenotypes by inhibiting DNA damage checkpoints and blocking B-cell terminal differentiation [20]. Normally, most HSCs are in a resting state. Only a small number can be used to maintain the hematopoietic balance by proliferating and differentiating. However, when hematopoietic cells are depleted or damaged, or under the action of an HSC mobilization agent or some cytokines, HSCs will significantly proliferate and more of them will enter the cell cycle to maintain hematopoiesis of the body [21, 22]. The overexpression of *BCL6* in cells obviously upset the physiological balance of HSCs. In addition, a large number of hematopoietic cells in RSS-SLD chickens were damaged and depleted, and HSCs were in a pathological state. The infinite proliferation of HSCs inhibits their maturation. These results indicate that the high expression of *BCL6* leads to excessive differentiation of MSCs into fat, which is one of the reasons for the increase in yellow bone marrow in RSS-SLD chicken bone marrow.

Overexpression of the *BCL6* inhibits chemotherapy-induced ROS production and apoptosis in B-cell lymphoma cells [23]. On the contrary, knockdown of the *BCL6* increases hypoxia-induced oxidative stress in cardiomyocytes [24]; our overexpression results are consistent with these findings. Excessive ROS levels can lead to cell senescence and death, while low levels are critical for maintaining the microenvironment of the stem cell pool. H_2_O_2_ in ROS plays an important role in the control of proliferation, differentiation, migration, and activation of signaling pathways [25]. Proto-oncogene *BCL6* belongs to the anti-apoptotic family, and *BCL6* acts as a transcriptional inhibitor. The target genes regulated by BCL6 are mainly related to cell activation, differentiation, and proliferation, and BCL6 can inhibit cell apoptosis by inhibiting transcription. Mitochondrial dysfunction and ROS accumulation can also promote apoptosis of cancer cells, and lead to the expression of the cell cycle inhibitor p53, cell cycle arrest in the G_2_/M phase, DNA fragmentation, and apoptosis induction [26]. Chemotherapy drugs such as cyclophosphamide can increase lipid peroxidation and apoptosis induced by ROS in sarcoma-180 tumor tissues [27]. In our study, the overexpression of *BCL6* decreased the ROS content of HSCs, which promoted the proliferation of HSCs and inhibited their apoptosis.

However, the results of mitochondrial function *in vivo* are contrary to those *in vitro*. There is evidence demonstrated that the cells with high ΔΨm were prone to continue to divide and form tumors, while low ΔΨm is conducive to the differentiation of embryonic stem cell transplantation mouse stem cells *in vivo* [28]. In our experiments, ΔΨm increased after *BCL6* overexpression *in vitro*, confirming the previous finding that overexpression of *BCL6* can lead to the oncogenic function of HSCs. On the contrary, the decrease of ΔΨm in RSS-SLD chicken may indicate *BCL6* has a negative effect on mitochondrial function under normal physiological conditions *in vivo*. Furthermore, the increase in ΔΨm generally promotes the increase in ATP. In the process of promoting cell proliferation, a large amount of ATP is also produced. Consistently, our results showed that the increase of ΔΨm and ATP along with the proliferation of HSCs after *BCL6* overexpression *in vitro*. On the other hand, we did not determine whether the ATP came from anaerobic respiration or from normal mitochondrial metabolism. Therefore, we speculated that abnormal proliferation of HSCs after *BCL6* overexpression *in vitro* may be similar to the production of lymphoma, which is powered by anaerobic respiration. This may also be the reason for the discrepant results between *in vitro* and *in vivo* experiments, indicating that BCL6 may exert different regulatory mechanisms on mitochondrial function *in vivo* and *in vitro*.

There are few reports of the effects of *BCL6* on HGFs and the specific mechanism is not clear. In our experiments, overexpression of the *BCL6* inhibited the level of HGFs and decreased the expression of hemoglobin α_1_ and hemoglobin β, similar to the results of RSS-SLD chickens. As a cell suppressor, BCL6 can specifically bind to the promoter DNA of the target gene, thereby inhibiting the transcription of the target gene [29].

## Conclusions

In conclusion, different degrees of mitochondrial dysfunction and HSCs differentiation disorder were observed in RSS-SLD chickens and SLD chickens. Studies at the cellular level found that overexpression of *BCL6* would lead to abnormal mitochondrial function, hematopoietic disorder, excessive proliferation, apoptosis inhibition, and a series of cancerous phenomena, indicating that the high expression of *BCL6* could inhibit the differentiation and development of HSCs by affecting the mitochondrial function, and might affect the growth and development of chickens.

## Data Availability

All data generated or analyzed during this study are available from the corresponding author on reasonable request.

## Abbreviations

BCL6: B-cell CLL/lymphoma 6
DCF: Dichlorofluorescein
HSCs: Hematopoietic stem cells
HGFs: Hematopoietic growth factors
MSCs: Mesenchymal stem cells
ΔΨm: Mitochondrial membrane potential
nDNA: Nuclear DNA
PCR: Polymerase chain reaction
qRT-PCR: Quantitative real-time PCR
RIPA: Radio immune precipitation assay
RSS: Runting and Stunting Syndrome
ROS: Reactive oxygen species
SLD: Sex-linked dwarf
